# Informed consent in genomic research and biobanking: taking feedback of findings seriously

**DOI:** 10.1080/11287462.2020.1717896

**Published:** 2020-02-23

**Authors:** Paulina Tindana, Cornelius Depuur, Jantina de Vries, Janet Seeley, Michael Parker

**Affiliations:** a Department of Health Policy, Planning and Management, University of Ghana School of Public Health, Accra, Ghana; b Navrongo Health Research Centre, Navrongo, Ghana; c University of Cape Town, Cape Town, South Africa; d MRC/UVRI Uganda Research Unit on Aids, Kampala, Uganda; e University of Oxford, Oxford, UK

**Keywords:** Broad consent, Africa, sample sharing, data sharing, genomic research feedback of findings

## Abstract

Genomic research and biobanking present several ethical, social and cultural challenges, particularly when conducted in settings with limited scientific research capacity. One of these challenges is determining the model of consent that should support the sharing of human biological samples and data in the context of international collaborative research. In this paper, we report on the views of key research stakeholders in Ghana on what should count as good ethical practice when seeking consent for genomic research and biobanking in Africa. This study was part of a multi-country qualitative case study conducted in three African countries: Ghana, Uganda and Zambia under the auspices of the Human Heredity and Health in Africa initiative (H3Africa). Our study suggests that while participants are willing to give consent for their samples and associated data to be used for future research purposes, they expect to receive feedback about the progress of the research and about the kinds of research being undertaken on their samples and data. These expectations need to be anticipated and discussed during the consent process which should be seen as part of an ongoing communication process throughout the research process.

## Introduction

In recent years, the key ethical challenges in the review and conduct of genomic research and biobanking in Africa have received considerable attention (de Vries et al., 2011; Marshall et al., 2006; Nyika, 2009; Parker & Bull, 2009). One of the most important of these is the question of which model of consent is most appropriate for decisions about future reuse of human biological samples and associated data in the context of international collaborative research (Igbe & Adebamowo, 2012; Tindana et al., 2012; Traore et al., 2015; Wendler & Emanuel, 2002). The challenges associated with obtaining valid consent in human subjects research in general have been well documented in the literature (Mandava, Pace, Campbell, Emanuel, & Grady, 2012; Marshall, 2008; Molyneux, Peshu, & Marsh, 2004) and there is evidence to suggest that while consent is widely acknowledged as an important pillar for the ethical conduct of research with humans, applying the principles and practice of informed consent, as stipulated in existing international and national ethics guidelines, presents difficult challenges for the conduct of genomic research and biobanking (de Vries et al., 2011; Grady et al., 2015; Wright, Koornhof, Adeyemo, & Tiffin, 2013).

A review of empirical studies on broad consent has suggested that there is a growing acceptance of broad consent, i.e. consent obtained at the time of sample collection for future research purposes, as a model for future research with human biological samples (Tindana & de Vries, 2016). This is based on the challenges with seeking specific consent where it is not possible to specify all future research uses in detail or where reconsenting participants is very difficult. There is also recognition that to be ethically acceptable, broad consent should be supported by appropriate governance structures to increase the utility of the samples and allow important scientific projects to be undertaken while respecting the rights and protecting the interests of research participants and their communities (Grady et al., 2015; Tindana & de Vries, 2016).

Questions, however, remain about how acceptable the concept of broad consent is and what counts as best practice when seeking consent for novel scientific projects such as genomics and biobanking in Africa. Two authors of this paper have previously suggested that seeking broad consent for future uses of samples is a form of entrustment and that certain conditions such as community engagement ought to be in place to complement broad consent (Tindana, Molyneux, Bull, & Parker, 2017). The concept of dynamic consent has also been proposed as a model of consent that uses information technology (IT) to facilitate engagement with participants and sample donors and enable them to consent to new research projects over a period of time (Kaye et al., 2015; Stein et al., 2013). However, this technology-based model of consent could face challenges in resource constrained settings, particularly in Africa.

We conducted a multi-country case study in Ghana, Uganda and Zambia under the auspices of the Human Heredity and Health in Africa initiative (H3Africa) to explore key stakeholders’ perspectives on good ethical practice for the review and conduct of genomics and biobanking in Africa. This paper reports on the Ghana case study and is based on an analysis of key stakeholders’ views on seeking consent for genomics and biobanking in international collaborative genomic research.

## Methods

The Ghana case study for this three-country study involved document reviews, observation of consent processes, focus group discussions, individual interviews and deliberative workshops with key stakeholders. While indepth interviews and focus group discussions are rather standard in qualitative research and hardly requires introduction, deliberative workshops are more novel and have not often been used in this kind of work. Drawing upon deliberative approaches to empirical ethics and deliberative democracy (Abelson et al., 2003; Marsh et al., 2013; O'Doherty, Hawkins, & Burgess, 2012; Parker, 2002), this method involves providing participants with the facts about a particular issue including the various views and perspectives about the issue. Then, based on this, participants are asked to reflect and identify the ethical issues and suggest possible solutions for addressing them. The goal is to arrive at a consensus on what should count as good ethical practice. The long process of deliberation is what distinguishes this method from traditional focus group discussions.

## Study setting

The study was conducted in the Kassena-Nankana districts of northern Ghana which hosts the Navrongo Health Research Centre (NHRC), a research institution that has been involved in international research collaborations for over 30 years (Oduro et al., 2012). The community members in these districts are familiar with genomic research. Three major genomic studies have been conducted in the area in the past, namely the MalariaGen project from 2005 to 2009 (MalariaGEN, 2008), a pathogen genomic research, and a collaborative genomic study between the Wits Health Consortium and InDepth Network focusing on cardiometabolic disease under the auspices of the Human Heredity and Health in Africa (H3Africa) initiative (H3Africa Consortium, 2014; Ramsay et al., 2016). Previous empirical ethics studies have focused on malaria genomic studies involving young children (Tindana et al., 2012; Tindana, Molyneux, & Bull, 2014) and explored the perspectives of parents and other stakeholders. This study was conducted between 2015 and 2016 and focused on the genomics of non-communicable diseases involving research participants between the ages of 40 and 60 years. We were also interested in getting a better understanding of how the challenges with consent identified in previous studies have evolved over the years. This research was approved by the Navrongo Health Research Centre Institutional Review Board (no. NHRCIRB206), the Ghana Health Service Ethical Review Committee (No.GHS-ERC-GM 07/02/16) and the Oxford University research ethics committee (OxTREC Reference: 580–16). Written informed consent was obtained from all the stakeholders interviewed.

### Sampling and data collection

Three categories of stakeholders were purposively selected for interviews as outlined in Table 1.

**Table 1.** Sample of participants.

Stakeholder group	Description	Type of interview	Number of interviews
Implementers of research	Genomic researchers	IDIs	2
	Fieldworkers	IDIs	1
	Field coordinator	IDIs	1
	Laboratory staff	FGDs	1 (6 discussants)
Reviewers of research	Members of research ethics committees	Deliberative workshops	2 (8 participants in each workshop)
Research participants and community members	AWI-Gen participants and those who refused	IDIs and FGDs	17 IDIs 12 FGDs

Stakeholder group 1 included implementers of research. We approached all the genomic researchers, fieldworkers, study coordinator and laboratory technicians involved in the AWI-Gen study for interviews. Stakeholder group 2 were members of research ethics committees who reviewed H3Africa research protocols in Ghana. An invitation was extended to all the members of the committees through their administrators and a convenient date for the committees was agreed upon. Stakeholder group 3 included AWI-Gen study participants and those who refused to take part in the genomic study. While the selection of groups 1 and 2 was quite simple, the selection for the third group of participants was more complex.

The AWI-Gen project recruited 2000 individuals between ages 40 and 60 years from four geographic zones of the Kassena-Nankana districts (North, South, East and West). We randomly selected two of these zones (East and West) for the Ethics project. With a list of participants obtained from the research team, fifty (50) AWI-Gen participants from each zone were selected and contacted individually in their homes for indepth interviews and focus group discussions. The project also had a record of community members who had refused to take part in the genomic study. Ten (10) of these community members were randomly selected and approached for interviews. Of these, three (3) refused to participate in the Ethics project.

## Data collection

### Document review

The document review involved the collection and review of institutional and national ethics guidelines and regulations governing health research in general with a focus on the requirements for conducting genomic research and biobanking in Ghana. Themes identified through this analysis were explored through indepth interviews with key stakeholders (members of ethics committees, genomic researchers, fieldworkers and research participants) on what should count as good ethical practice.

### Participant observation during the consent process

This study was conducted in relation to the third genomic study to be conducted in the Kassena-Nankana districts in 2016, namely the H3Africa AWI-Gen project which focused on cardiometabolic diseases (Ramsay et al., 2016). This project utilised a three-stage consent process: an initial contact with potential participants at the community level to inform them about the study and to invite interested and willing participants to the recruitment centre for further discussions; group discussions with all potential participants reporting at the recruitment centre where the project is explained to them; and finally one-on-one individual informed consent in a separate room where participants make a decision to either participate in the research or decline. Those who consent thumbprint or sign the consent forms.

In the ethics project reported here, the Ghana study team sought verbal consent from the AWI-Gen study team and participants to observe the group consent sessions at the recruitment centre. Given that the disclosure of information is one of the key elements of informed consent, we sought to gain a better understanding of how information about the genomic study was shared with the participants and the questions raised by participants. Notes were taken on how the genomic concepts of the project were explained in the local languages including the use of sample collection tubes to explain the amount of blood to be obtained from the participants. Only two group discussions were observed and no names or personal information of the participants were recorded.

### Indepth interviews, focus group discussions and deliberative workshops

A total of 126 people participated in this study; 21 individual interviews (2 researchers, 2 fieldstaff, 10 genomic research participants and 7 refusers), 14 focus group discussions (involving an average of 6 people in each group) and 2 deliberative workshops with members of 2 research ethics committees (involving 8 REC members in each group). The topics discussed during these indepth interviews and group discussions focused on general views on genomics research, understanding of the rationale for sample and data sharing, views on broad consent and other models of consent and recommendations for good ethical practice. The individual interviews lasted for an average of 45 min while the FGDs were about an hour.

The deliberative workshops were conducted with members of two research ethics committees in Ghana. This involved a formal presentation on the ethical issues in genomic research, the challenges with consent and the pros and cons of current proposed consent models such as broad and dynamic consent. Following the presentation of these materials, participants were asked to discuss selected issues and provide practical solutions for the way forward. These discussions were recorded in minutes, which were used to inform the formal analysis of the data obtained in these deliberative workshops. The workshops followed on from the IDIs and FGDs and thus built on some of the themes that emerged from these interviews.

## Data management and analysis

All the interviews and focus group discussions were audio-recorded except one of the deliberative workshops with the research ethics committees, where the members collectively declined audio recording. Detailed notes were taken during this meeting and formed part of the data analysis. All the focus group discussions and individual interviews with the community members were conducted in the local languages (Kasem and Nankani). These were translated into English during the transcription process by two research assistants.

Data analysis was ongoing throughout the study, using thematic content analysis. Guided by the objectives of the research, we developed a coding scheme to facilitate the data analysis, aided by the qualitative analysis software, NVivo 10. We explored themes around general understanding of genomics among the research participants, views on the consent process used in the genomic research study and recommendations on how these processes could be improved for future studies. We also paid particular attention to new issues emerging from the data such as the importance of feedback of research results.

## Results

The results of the document reviews will be reported in a seperate manuscript. This paper focuses on the key overarching themes that emerged from the interviews and focus group discussions. These include understanding and recall of the genomic study; the process of disclosure of information, acceptability of broad consent for sample and data sharing; and expectations on feedback of findings. This paper discusses these themes in turn with extracts from the interviews used to support these findings.

### General understanding and recall of genomic research

Valid consent requires that potential participants understand the research and voluntarily agree to participate or decline participation. To elicit views on what will count as good ethical practice when seeking consent to genomics and biobanking, it was important to explore stakeholders’ general knowledge about key concepts in genomics and biobanking such as the rationale for sample and data sharing. The interviews with genomic research participants were conducted 6 months after their participation in the H3Africa genomic study and most were able to recall in detail each of the processes they were taken through during the project. They gave descriptions of what they were told about the project, the different tests and measurements that were conducted on them, the different samples (blood and urine) they provided and so forth.

She said that, … . there has been an increase in diseases like stroke and those related to the heart and therefore there was the need to conduct this study to find some treatment to those diseases. For this reason, they needed to take our blood samples to carry out some test so they could study those diseases. (FGD with women)

Most participants also recalled that the project was a collaborative study involving several countries including Ghana and that the samples and data would be sent to South Africa for further analysis and shared with other scientists.

We were also told that some of the blood samples will be kept here [in Navrongo]and some too will be sent abroad for testing but the results of the blood sent abroad will not be ready now, it will take some time. (FGD with women, EAST)

Two local terms for genes emerged during the interviews: “*nkurugu*” in Nankani and “*Nyinyogo*” for Kasem. These terms were used to describe inherited traits that are passed on from one generation to another in the two local languages:

It is difficult to explain what “nkurugu” is in our local language unlike in English or Akan, but what I know is that it means what you have inherited from your parents and therefore take after their bloodline and their likeness. It has to do with inheritance from generations in your family. That is my understanding. (FGD with male genomic research participant)

Some participants in the focus group discussions explained that there are certain diseases that run through families which suggested that they have some general understanding of the concept of heredity and health, which is important in the conduct of genomic research. It is noteworthy that some participants mentioned diseases that are not familial inherited diseases or what would normally be referred to as “genetic diseases” as demonstrated in the quote below:

All these diseases they’ve mentioned; epilepsy, leprosy and tuberculosis are diseases that can be found in the blood and can be transferred or inherited through a family line. As long as you belong to a family that has any of these diseases, the likelihood of a member from that same family getting such a disease high. It doesn’t matter the generation of such a family, someone will inherit it. (FGD with female genomic research participants)

As noted in our observation of the consent process described earlier, many of the community members we interviewed did not raise questions about other aspects of the genomic study such as data and sample sharing.

### Addressing local sensitivities to blood sampling

Local communities’ sensitivities to blood sampling have been well documented in the literature (Beidelman, 1963; Fairhead, Leach, & Small, 2006; Molyneux & Geissler, 2008; Tindana et al., 2014) and previous studies conducted in Kassena-Nankana districts have also highlighted community members’ apprehensions about the collection of blood samples for research purposes (Tindana et al., 2012, 2014). Not surprisingly, these were also of concern to research participants in the present study. According to the field staff we interviewed, residents in the local community often question the rationale for collecting blood from an individual for research purposes and the implications of such procedures on the health of that individual. Group discussions with the field staff also suggested that even when participants have agreed to take part in the study and report to the recruitment centre, they become agitated during the blood sampling process.

You know we used these tubes, about five tubes for the blood. And in the local language they will refer to these tubes as “Kolma” in Nankam or “Salma” in Kasem which is similar to a beer bottle … , so when you go [to the community] to invite them it’s not always easy but when they come, they see that the tubes are small. When you want to interpret the “tube” to them in the local language, you have to use the word “bottle” and bottle means “the big bottle” but the local people too will take it to mean “beer bottle”. (Fieldworker, male)

These claims by field staff about community views on the collection of tubes of blood were confirmed by our interviews with the research participants themselves, as illustrated in the quote below:

They made us lie down in a room and they run some checks on us with a machine […]. But before that, we were given some forms. We later on went into a room where blood was taken. 3 bottles were taken from some people, others 4 and others also had 5 bottles taken. At that point, some of us got upset because we felt that if they wanted to carry out any test; they would need just 1 bottle to do that, not 3, 4 or 5 bottles … (FGD with genomic research participant)

Field staff who handled the blood sampling process during the recruitment also gave narratives about some cultural beliefs and implications of collecting blood from community members who are strongly rooted in a more traditional way of life, which includes beliefs in the power of amulets. For instance, one laboratory staff member related the following story:

We had one man who willingly gave out his hand [for blood sampling], then when we prepared the hand and wanted to go into the vein he asked me if I have excused him? Then I said oh I did that, maybe you didn’t hear. Then he said no, I didn’t. Well I can go ahead; I tried even the second time and I failed, then he said [it is ]because I did not inform him or excuse him in a manner that he will understand. So I was wondering how I was supposed to put it, then, he himself told me that I should say “gafara” I want to pick blood from him. And when I did, I was able to draw the blood. So, after drawing the blood he explained to me how it should be done. He was wearing a whole lot of [traditional] things on his body! (FGD with research staff)

The *gafara* referred to in the above narrative means “excuse me” and the oldman was essentially telling the research staff the importance of expressing this traditional way of seeking permission before drawing blood samples. Some of the laboratory staff we interviewed admitted that they did not speak the local language of the research participants and thus when issues were raised at the point of sample collection, they were unable to respond appropriately due to this barrier in communication. Surprisingly, none of the research participants we interviewed mentioned barrier in communication as a challenge in the consent process.

### Views on seeking consent for biobanks

In seeking the views of the study participants on the concept and practice of broad consent, we needed to explain what the concept is, why it has been proposed as a model for genomics and biobanking and the other alternative models of consent such as blanket consent, multilayered, tiered consent and dynamic consent. There was general acceptance of the broad consent model by most of the stakeholders we interviewed including among the research staff and genomic research participants.

It wouldn’t be an issue since you people are the ones doing the work and know what is best. If such samples are useful, you can go ahead to use them for our benefit as well as yours. (IDI with female genomic research participant 02)

However, other respondents, particularly the REC members and researchers were of the view that efforts should be made to keep participants and community members updated on the status of research conducted on these stored samples, as a way of sustaining a trust relationship between the research team and the local communities and allowing some control over these samples.

the participants should know exactly what the samples are likely to be used for in the near future. So, if it’s broad at least, let the participants know the kind of research or the kind of studies that the samples will be used for in future. So maybe if its malaria research, maybe sickle cell research or something else just be clear about that but I think it’s somehow similar to the second type, multi-layered consent. (group discussions with RECs 01)

Discussions on broad consent also raised issues about the feasibility of using stored samples for future studies. Some participants could not comprehend that it is possible for samples to be stored for many years and be used for research that can provide any meaningful information.

In my opinion, I would rather prefer they come back and take new blood samples to work with rather than use the old samples. To me, such samples could go bad and no matter how it is preserved, it might not be as potent as the fresh blood, such stored samples are expired. Laughing! (FGD with male genomic research participant)

Most REC members were, however, skeptical about the use of broad consent and recommended that this model should be reviewed with some caution. They suggested that each protocol should be reviewed based on its own merit and the use of broad consent should be justified. Thus broad consent should be the exception and not the rule.

If care is not taken, it will turn out that every form of consent being sought will be broad consent and that is not good, it should be considered based on the type of research work to be carried out. (group discussion with RECs 02)

These responses suggest that despite the growing recommendations in international guidelines on broad consent as a model for genomics and biobanks, participants expect the community – and themselves as individual participants – to receive feedback about the progress of the research and about the kinds of research being undertaken on their samples and data.

For me it is good to let us know; “when two people set up a trap for a prey, it is best for the same two to inspect the trap to see whether there is a catch or not”. If such reserved sample is still potent, it’s good to let us know before it is used for further studies. On the other hand, if the samples are spoilt, you still will need to inform us so we can provide new samples. (FGD with male genomic research participants)

The research ethics committees and research team were of the view that there is an urgent need for clear national and institutional ethical standards to guide the review and conduct of genomics and biobanking in Ghana. A national document should clearly stipulate that all genomic studies should be reviewed by a national or institutional ethics committee and for local research institutions to have control over the samples collected and exported for analysis.

The issue of researchers collecting blood samples and asking to store it for future use is becoming too common. There must be some regulation on this. (Discussant; group discussion with REC02)

there should be clear guidelines as to the limit of the use of the samples. How am I going to use the samples? To what extend can I use the samples? And if am using these samples, to whom do I go to? To whom do I go? Do I go to the national ethics bodies? Should I go back to the research participants? All these should be clearly spelt out in guidelines. (IDI with researcher 01)

Interviews with the researchers, field staff and members of the research ethics committees suggested that there is an important role for RECs in any governance framework for genomics and biobanking. They suggested that decisions about future uses of samples and data should involve the RECs that originally reviewed the proposal:

I have no problem with the sharing of the samples but it should be done in an ethically sound manner. And it should be done such that, whichever ethics committee that is going to grant approval for the sample to be used should always have a link with the mother group so that you are updated as to the extent to which your samples are being used. So if you have some objectives to make, you can, otherwise it will look as if you can just pick someone’s sample, take it somewhere and then manipulate it in whatever way because this is genes we are talking about so you can always manipulate genes anyhow. So if you don’t have a hand in it and it reaches a point whereby the sample can be used by anybody elsewhere, you can’t tell when things will go wrong. (DI with researcher 3, NHRC)

Surprisingly, most of the AWI-Gen research participants were not familiar with the work of the research ethics committees and thus could not provide recommendations on how RECs could be involved in governance issues:

I am aware that for every work that is to go on well, there must be a committee to oversee that work. What I do not know is the details of the committee’s work. (IDI with male participant, EAST)

### Taking feedback of research results seriously

An important theme that emerged from interviews with all the stakeholders was the importance of feedback of research results to participants and communities. During the course of the H3Africa AWI-Gen project, the results of the baseline assessments such as the height, weight and blood pressure were given to the participants. Some respondents in our ethics study expressed gratitude that they were referred to the hospital for treatment when their test results were outside the normal values. There was, however, an expectation that further tests conducted on the samples would be shared with participants, although they did not explicitly indicate whether the results they expected should include individual genetic results and/or general research results:

Yes, they told us they were going to take some of the blood samples abroad and investigate further about other illnesses we may have. So, we are still waiting for feedback from them. (IDI with female genomic research participant 03)

As suggested in the above extracts, the expectations for feedback from these participants are based not only on the desire for knowledge on their health status but also a fulfilment of an assurance given by the research team. There were clear indications from these interviews that the field staff seeking consent had given some assurances that efforts would be made to provide feedback at the end of the project and this had raised participants’ expectations.

The issue of feedback of results was such a strong theme throughout the interviews that it emerged as one of the main reasons why some members of the local community refused to participate in the genomic study. According to the fieldworkers who conducted the consent process for the project, there is some dissatisfaction among some sections of the community about the lack of feedback from previous studies involving sample collection. These views were confirmed in our interviews with “refusers” in the various communities:

they ever came and took blood samples for a similar purpose and did not come back to tell us anything about the blood they took nor the screening they did. I told them I was no longer interested and for that matter I was not prepared to go this time around. Whether the first time they took the blood and sold or they discovered some diseases, they never came back. Under this circumstance, why would I be interested in participating again? I need the blood in my veins to be able to work. (IDI with a refuser 05)

I know that the quantity [of blood], is small but the fact that the first group did not come back to tell us what they used our blood for or whether they discovered any diseases, has discouraged me. (IDI with refuser 02)

This lack of feedback from previous studies has discouraged some members of the community from participating in research and getting involved in any activities related to research. These responses highlight the importance of feedback to research participants and the need for researchers to take this seriously in the research process. Feedback was also seen as a way of closing the study so that participants do not assume that the study is still ongoing. In addition, the feedback process was seen as a way to empower members of the community with knowledge about their health status.

Researchers and laboratory personnel were of the view that while feedback of results is important, care needs to be taken on the nature of the feedback and how that information is handled. In reference to the population structure component of the project, where family trios (mother, father, child) were recruited, they suggested that issues about paternity were raised which suggested that such information is regarded as sensitive and best kept private:

I remember in the population structure aspect, some of them asked because we were taking trios, father, mother and child. So, some of them asked questions like, you know these have to do with paternity issues whether or not you’re going to find out that this person is my child, and all those things and actually that has been one of the main issues. (FGD with research staff)

You know there are some diseases or some conditions that are peculiar to certain families and they are such that they keep it secret. So, if you’re going to work on people’s blood, they begin to think that you’ll discover some of those secret conditions that are only known to the family and they don’t want people out there to get to know. (FGD with research staff)

It is worth stating that during the initial contact with the genomic research participants to take part in this ethics project, many of them brought out the consent forms they signed for the genomics project and asked if the ethics project team had come to disseminate the results from the genomic study.

## Discussion

Seeking valid consent is one of the key ethical challenges in the review and conduct of genomic research and biobanking globally. Recent revisions of international guidelines such as Council for International Organization of Medical Sciences (CIOMS) recommend broad informed consent as a model for research involving future uses of samples and associated data (CIOMS, 2016). However, there is still ongoing debate about its acceptability in Africa. The results of this study confirm that the challenges with seeking informed consent for genomic studies persist, even in communities that have been exposed to these types of studies for over a decade.

While it was clear that the genomic research participants we interviewed in this study had a general understanding of the various procedures involved in the genomic research project and expressed their willingness to participate, they also expressed some concerns about sample collection. This is consistent with previous empirical studies conducted in this study setting (Tindana et al., 2012, 2014) and other research settings in Africa (Beidelman, 1963; Fairhead et al., 2006; Molyneux & Geissler, 2008). The fact that these concerns persist even after engagement processes within the community suggest that research projects need to anticipate these local cultural sensitivities around blood sample collection and address them more pro-actively during the community engagement and informed consent process. Researchers should use innovative ways of explaining the quantity of blood collected for research process. This could involve, taking the blood collecting tubes to the community and demonstrating how the actual sampling process is conducted to demystify the process.

The study has also highlighted the need for consent to be seen as a communicative process throughout the research process, including at the point of sample collection. While this is recommended for all types of studies, there is very limited data on how often researchers engage with participants beyond the initial consent process including the sample collection stage. Given the complexities in genomic research and the communication barriers highlighted by the laboratory staff, there is the need to strengthen the cultural competence and language skills of research staff who interact directly with research participants at various stages of the research process including those involved in sample collection.

Feedback of research findings emerged as an important theme both in our observation of the consent process and the interviews and focus group discussions conducted with the various stakeholders. There is a strong expectation from research participants that the results of the genomic study including the implication of these results, would be shared with them.

There are several factors that could be contributing to these community expectations for feedback. The H3Africa genomics project recruited healthy adults, who had donated blood samples for studies on cardiometabolic disease. This appears to have raised expectations from the participants on knowing the results of the various tests conducted on the samples and the implications on their health. There was therefore some evidence of a “diagnostic misconception” where participants assumed that the collection of samples for research is for diagnostic purposes, as reported in other H3Africa studies in South Africa (Kutschenko, 2012; Masiye, Mayosi, & de Vries, 2017). Second, in the process of seeking consent, field staff had made assurances to participants that results would be shared with them. These promises were also made during the group consent processes we observed. The latter highlights the need for staff seeking consent to be cautious about making promises that may not be fulfilled during the research process. While there is extensive literature on feedback of individual genetic research findings including how to handle incidental findings (Dixon-Woods, Tarrant, Jackson, Jones, & Kenyon, 2011; Fernandez, Kodish, & Weijer, 2003; Knoppers, Deschênes, Zawati, & Tassé, 2013; MacNeil & Fernandez, 2006), discussions on these issues have only evolved recently within the H3Africa Consortium in Africa (H3Africa Feedback of Findings Policy ).

The literature suggests that in the context of genomics and biobanking, there are different kinds of research results that can be returned to research participants. Knoppers et al have classified these findings as follows: *Baseline assessments* which include measurements such as blood pressure, lung function, bone density, height, weight, fat, and others are taken at baseline or at any subsequent assessment; *Individual genetic findings* are those discovered during research, which concerns an individual participant, and have potential health or reproductive impact; *Incidental findings* are unforeseen findings concerning a research participant that has potential health or reproductive importance. These are discovered in the course of the research but are outside its objectives. *General research results* refer to aggregate results drawn from the analysis of data and samples of a group of research participants (Fernandez et al., 2003; Knoppers et al., 2013). The H3Africa guideline for the return of individual genetic finding recommends that return of individual genetic findings should proceed with caution and should follow a careful consideration of key principles. This includes assessing “whether the particular finding holds value for the individual” and ensuring that “patients are appropriately informed of the implications of the findings” (H3Africa Feedback of Findings Policy ). Further studies should be encouraged to clarify which of these types of findings are considered valuable to research participants and communities and how such findings should be communicated.

In the context of biobanks, empirical studies have suggested that despite the growing acceptance of broad consent for genomics and biobanking, there is also an expectation from research participants and sample donors for some feedback on the range of projects that draw on their samples and data. Increasingly, authors have argued that researchers have an ethical obligation to share research results to participants based on the principles of respect, beneficence and reciprocity (Dixon-Woods et al., 2011; Fernandez et al., 2003; Knoppers et al., 2013; MacNeil & Fernandez, 2006). Communicating research results does not only demonstrate respect to participants but is also a way of acknowledging their important contribution to science. Feedback is also about sharing knowledge which could help participants and their communities live healthier lifestyles which could contribute to positive health outcomes. For example, Kerasidou has proposed that communicating aggregate findings from genomic studies should be perceived as “sharing knowledge” and not just “returning results”. She argues that the dissemination of “aggregate ﬁndings as sharing of knowledge not only describes the exercise more accurately but can also help mitigate some of the risks involved with returning non-individual aggregate results” (Kerasidou, 2015). A key challenge to sharing such aggregate knowledge is finding the appropriate methods and how the views of communities ought to be incorporated into policies guiding decisions on when, how and what to feedback to research participants and communities participating in genomic studies. Further exploratory studies will be helpful to unpack these issues and provide recommendations on how research results could be shared with individuals, groups and communities.

It is interesting that although there is support for the use of broad consent for sample and data sharing, participants in our study described a desire for information on the use of these samples, the results of the tests conducted and the implications on their health. These responses including those from members of the research ethics committees suggest that participants should be regularly updated about the status of their samples.

Dynamic consent has been proposed as a model of consent that uses information technology (IT) to enable participants to consent to new research projects over a period of time (Kaye et al., 2015; Stein & Terry, 2013; Steinsbekk, Myskja, & Solberg, 2013). Whilst this has a number of limitations as an approach to consent, and the technology required for this model will present challenges in these rural African settings, the idea of a dynamic platform for ongoing communication between participants and researchers about research being undertaken and uses of samples, is appealing for its potential to increase conﬁdence and trust in the research and researchers. We suggest that future research should explore possibilities for combining technology-based approaches to include more traditional methods of communication such as community durbars [gatherings] and meetings to facilitate feedback of research results to participants and ongoing communication with research participants and communities.

## Conclusion

Seeking consent for genomic research and biobanking remains one of the key ethical challenges in international collaborative genomic research. Our study suggests that what would count as good ethical practice is an approach in which consent is seen as part of an ongoing communication process throughout the research process; where there is regular feedback of study findings to the participants; and where participants have some control over what will happen to their samples and data. Participants’ high expectation for feedback is likely to affect the conduct of future genomics studies in these communities if these expectations are not met. We encourage further empirical ethics studies to identify best practices for sharing genomic research results to research participants and communities.

## References

[CIT0001] Abelson, J., Forest, P. G., Eyles, J., Smith, P., Martin, E., & Gauvin, F. P. (2003). Deliberations about deliberative methods: Issues in the design and evaluation of public participation processes. *Social Science & Medicine*, *57*(2), 239–251. 12765705 10.1016/s0277-9536(02)00343-x

[CIT0002] Beidelman, T. O. (1963). The blood covenant and the concept of blood in Ukaguru. *Africa: Journal of the International African Institute*, *33*(4), 321–342.

[CIT0003] CIOMS. (2016). *International ethical guidelines for health-related research involving humans* (4th ed.). Geneva: Council for International Organizations of Medical Sciences (CIOMS). 40523065

[CIT0004] de Vries, J., Bull, S. J., Doumbo, O., Ibrahim, M., Mercereau-Puijalon, O., Kwiatkowski, D., & Parker, M. (2011). Ethical issues in human genomics research in developing countries. *BMC Medical Ethics*, *12*, 5. 21418562 10.1186/1472-6939-12-5 PMC3076260

[CIT0005] Dixon-Woods, M., Tarrant, C., Jackson, C. J., Jones, D. R., & Kenyon, S. (2011). Providing the results of research to participants: A mixed-method study of the beneﬁts and challenges of a consultative approach. *Clinical Trials: Journal of the Society for Clinical Trials*, *8*, 330–341. 10.1177/1740774511403514 21730081

[CIT0006] Fairhead, J., Leach, M., & Small, M. (2006). Where techno-science meets poverty: Medical research and the economy of blood in The Gambia, West Africa. *Social Science & Medicine*, *63*(4), 1109–1120. 16630676 10.1016/j.socscimed.2006.02.018

[CIT0007] Fernandez, C. V., Kodish, E., & Weijer, C. (2003). Informing study participants of research results: An ethical imperative. *IRB: Ethics and Human Research*, *25*, 12–19. 14569989

[CIT0008] Grady, C., Eckstein, L., Berkman, B. E., Brock, D., Cook-Deegan, R., Fullerton, S. M., … Wendler, D. (2015). Broad consent for research with biological samples: Workshop conclusions. *AJOB*, *15*(9), 34–42. 10.1080/15265161.2015.1062162 PMC4791589 26305750

[CIT0009] H3Africa Consortium. (2014). Enabling the genomic revolution in Africa. *Science*, *344*, 1346–1348. 24948725 10.1126/science.1251546 PMC4138491

[CIT0010] H3Africa Feedback of Findings Policy. Retrieved from https://h3africa.org/wp-content/uploads/2018/05/H3Africa20Feedback20of20Individual20Genetic20Results20Policy.pdf

[CIT0011] Igbe, M. A., & Adebamowo, C. A. (2012). Qualitative study of knowledge and attitudes to biobanking among lay persons in Nigeria. *BMC Medical Ethics*, *13*(1), 27. 23072321 10.1186/1472-6939-13-27 PMC3507723

[CIT0012] Kaye, J., Whitley, E. A., Lund, D., Morrison, M., Teare, H., & Melham, K. (2015). Dynamic consent: A patient interface for twenty-first century research networks. *European Journal of Human Genetics*, *23*(2), 141–146. 24801761 10.1038/ejhg.2014.71 PMC4130658

[CIT0013] Kerasidou, A. (2015). Sharing the knowledge: Sharing aggregate genomic findings with research participants in developing countries. *Developing World Bioethics*, *15*(3), 267–274. 25292263 10.1111/dewb.12071 PMC4632193

[CIT0014] Knoppers, B. M., Deschênes, M., Zawati, M. H., & Tassé, A. M. (2013). Population studies: Return of research results and incidental findings Policy Statement. *European Journal of Human Genetics*, *21*, 245–247. 22781095 10.1038/ejhg.2012.152 PMC3573193

[CIT0015] Kutschenko, L. (2012). Diagnostic misconceptions? A closer look at clinical research on Alzheimer’s disease. *Journal of Medical Ethics*, *38*, 57–59. 21873309 10.1136/medethics-2011-100026

[CIT0016] MacNeil, S. D., & Fernandez, C. V. (2006). Informing research participants of research results: Analysis of Canadian university based research ethics board policies. *Journal of Medical Ethics*, *32*, 49–54. 16373524 10.1136/jme.2004.010629 PMC2563276

[CIT0017] MalariaGEN. (2008). A global network for investigating the genomic epidemiology of malaria. *Nature*, *456*(7223), 732–737. 19079050 10.1038/nature07632 PMC3758999

[CIT0018] Mandava, A., Pace, C., Campbell, B., Emanuel, E., & Grady, C. (2012). The quality of informed consent: Mapping the landscape. A review of empirical data from developing and developed countries. *Journal of Medical Ethics*, *38*(6), 356–365. 22313664 10.1136/medethics-2011-100178 PMC4825806

[CIT0019] Marsh, V., Kombe, F., Fitzpatrick, R., Williams, T. N., Parker, M., & Molyneux, S. (2013). Consulting communities on feedback of genetic findings in international health research: Sharing sickle cell disease and carrier information in coastal Kenya. *BMC Medical Ethics*, *14*(1), 41. 24125465 10.1186/1472-6939-14-41 PMC4016314

[CIT0020] Marshall, P. A. (2008). “Cultural competence” and informed consent in international health research. *Cambridge Quarterly of Healthcare Ethics*, *17*(2), 206–215. 18312735 10.1017/S0963180108080237

[CIT0021] Marshall, P. A., Adebamowo, C. A., Adeyemo, A. A., Ogundiran, T. O., Vekich, M., Strenski, T., … Rotimi, C. N. (2006). Voluntary participation and informed consent to international genetic research. *American Journal of Public Health*, *96*(11), 1989–1995. 17018820 10.2105/AJPH.2005.076232 PMC1751828

[CIT0022] Masiye, F., Mayosi, B., & de Vries, J. (2017). “I passed the test!” evidence of diagnostic misconception in the recruitment of population controls for an H3Africa genomic study in Cape Town, South Africa. *BMC Medical Ethics*, *18*(1), 12. 28202021 10.1186/s12910-017-0175-z PMC5311841

[CIT0023] Molyneux, S., & Geissler, P. W. (2008). Ethics and the ethnography of medical research in Africa. *Social Science & Medicine*, *67*(5), 685–695. 18455856 10.1016/j.socscimed.2008.02.023 PMC2682176

[CIT0024] Molyneux, C., Peshu, N., & Marsh, K. (2004). Understanding of informed consent in a lowincome setting: Three case studies from the Kenyan Coast. *Social Science and Medicine*, *59*(12), 2547–2559. 15474208 10.1016/j.socscimed.2004.03.037

[CIT0025] Nyika, A. (2009). Ethical and practical challenges surrounding genetic and genomic research in developing countries. *Acta Tropica*, *112*(Suppl. 1), S21–S31. 19665983 10.1016/j.actatropica.2009.07.034

[CIT0026] O'Doherty, K. C., Hawkins, A. K., & Burgess, M. M. (2012). Involving citizens in the ethics of biobank research: Informing institutional policy through structured public deliberation. *Social Science & Medicine*, *75*(9), 1604–1611. 22867865 10.1016/j.socscimed.2012.06.026

[CIT0027] Oduro, A. R., Wak, G., Azongo, D., Debpuur, C., Wontuo, P., Kondayire, F., … Binka, F. (2012). Profile of the navrongo health and demographic surveillance system. *International Journal of Epidemiology*, *41*(4), 968–976. 22933645 10.1093/ije/dys111

[CIT0028] Parker, M. (2002). A deliberative approach to bioethics. In K. Fulford, D. Dickenson, & T. Murray (Eds.), *Healthcare ethics and human values* (pp. 29–35). Oxford: Blackwell.

[CIT0029] Parker, M., & Bull, S. (2009). Ethics in collaborative global health research networks. *Clinical Ethics*, *4*, 165–168.

[CIT0030] Ramsay, M., Crowther, N., Tambo, E., Agongo, G., Baloyi, V., Dikotope, S., … Sankoh, O. (2016). H3Africa AWI-gen collaborative centre: A resource to study the interplay between genomic and environmental risk factors for cardiometabolic diseases in four sub-Saharan African countries. *Global Health, Epidemiology and Genomics*, *11*, 4–21. 10.1017/gheg.2016.17 PMC5732578 29276616

[CIT0031] Stein, D., & Terry, S. F. (2013). Reforming biobank consent policy: A necessary move away from broad consent toward dynamic consent. *Genetic Testing and Molecular Biomarkers*, *17*(12), 855–856. 24283583 10.1089/gtmb.2013.1550

[CIT0032] Steinsbekk, K. S., Myskja, B. K., & Solberg, B. (2013). Broad consent versus dynamic consent in biobank research: Is passive participation an ethical problem? *European Journal of Human Genetics*, *21*, 897–902. 23299918 10.1038/ejhg.2012.282 PMC3746258

[CIT0033] Tindana, P., Bull, S., Amenga-Etego, L., de Vries, J., Aborigo, R., Koram, K., … Parker, M. (2012). Seeking consent to genetic and genomic research in a rural Ghanaian setting: A qualitative study of the MalariaGEN experience. *BMC Medical Ethics*, *13*(1), 15. 22747883 10.1186/1472-6939-13-15 PMC3441464

[CIT0034] Tindana, P., & de Vries, J. (2016). Broad consent for genomic research and biobanking: Perspectives from low- and middle-income countries. *Annual Review of Genomics and Human Genetics*, *17*, 375–393. 10.1146/annurev-genom-083115-022456 26905784

[CIT0035] Tindana, P., Molyneux, C. S., & Bull, S. (2014). Ethical issues in the export, storage and reuse of human biological samples in biomedical research: Perspectives of key stakeholders in Ghana and Kenya. *BMC Medical Ethics*, *15*, 76. 25326753 10.1186/1472-6939-15-76 PMC4210627

[CIT0036] Tindana, P., Molyneux, S., Bull, S., & Parker, M. (2017). ‘It is an entrustment’: Broad consent for genomic research and biobanks in sub-Saharan Africa. *Developing World Bioeth*, *00*, 1–9. 10.1111/dewb.12178 PMC6624131 29063669

[CIT0037] Traore, K., Bull, S., Niare, A., Konate, S., Thera, M. A., Kwiatkowski, D., … Doumbo, O. K. (2015). Understandings of genomic research in developing countries: A qualitative study of the views of MalariaGEN participants in Mali. *BMC Medical Ethics*, *16*(1), 42. 26077875 10.1186/s12910-015-0035-7 PMC4469103

[CIT0038] Wendler, D., & Emanuel, E. (2002). The debate over research on stored biological samples: What do the sources think? *Archives of Internal Medicine*, *162*, 1457–1462. 12090881 10.1001/archinte.162.13.1457

[CIT0039] Wright, G. E., Koornhof, P. G. J., Adeyemo, A. A., & Tiffin, N. (2013). Ethical and legal implications of whole genome and whole exome sequencing in African populations. *BMC Medical Ethics*, *14*(1), 21. 23714101 10.1186/1472-6939-14-21 PMC3668248

